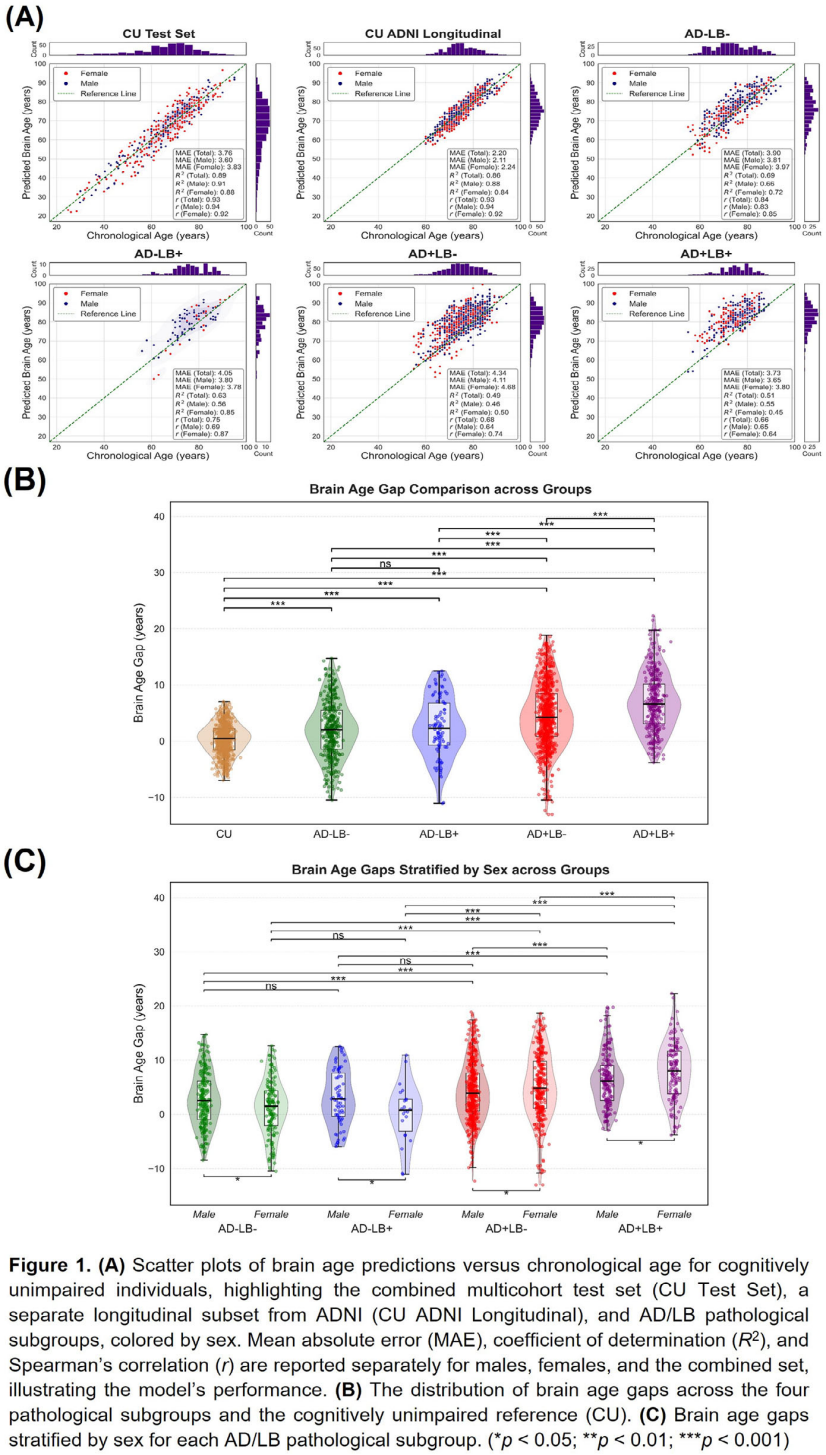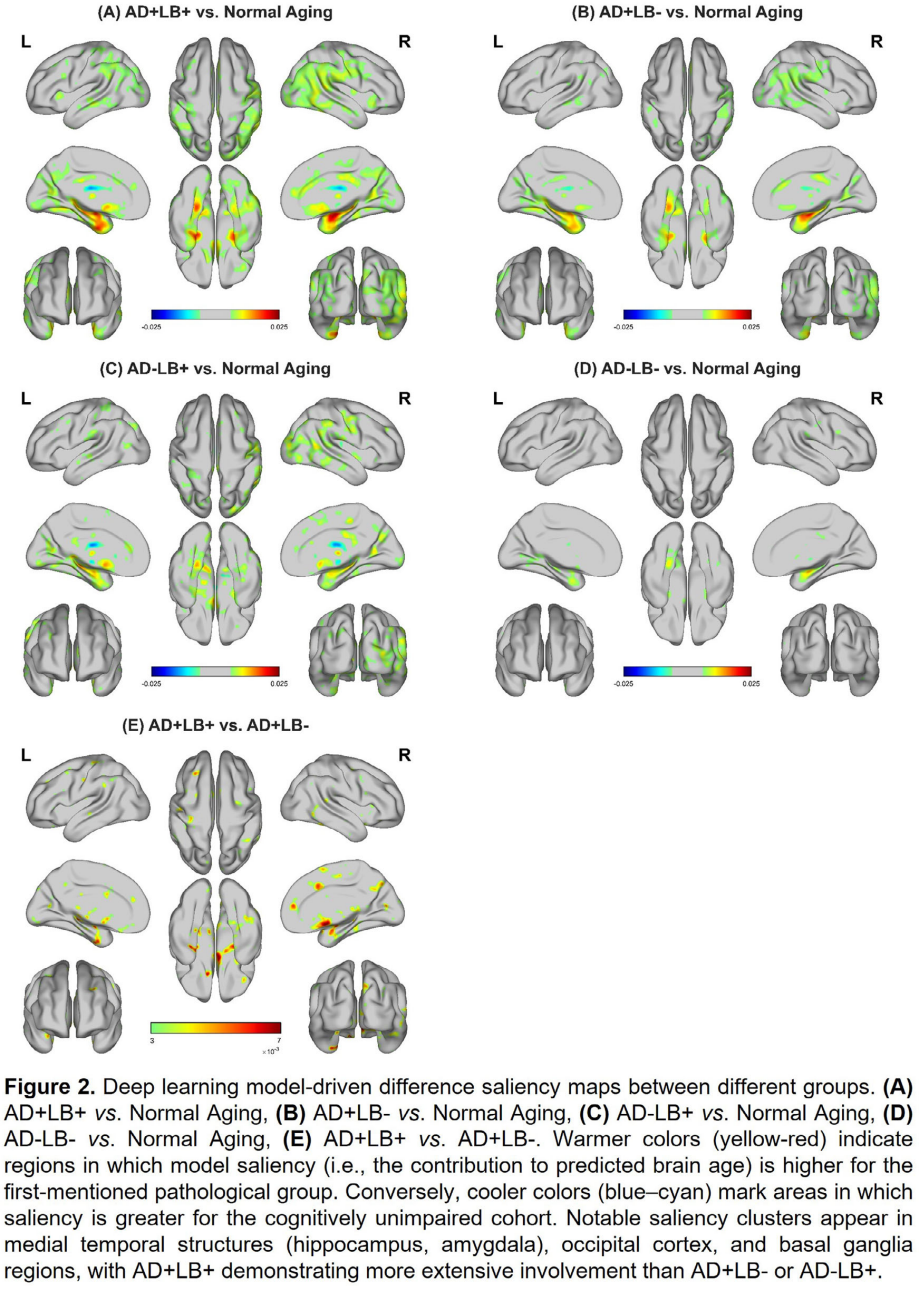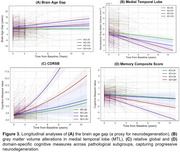# Synergistic Neurodegeneration in Alzheimer's Disease with Lewy Body Co‐Pathology: Insights from Deep Learning and CSF α‐Synuclein Seed Amplification Assays

**DOI:** 10.1002/alz70856_101717

**Published:** 2025-12-25

**Authors:** Babak Ahmadi, Zohreh Morshedizad, Hojjatollah Sadeqi, Breton M. Asken, Melissa J. Armstrong, Mostafa Reisi Gahrooei, Abbas Babajani‐Feremi

**Affiliations:** ^1^ University of Florida, Gainesville, FL, USA; ^2^ Fixel Institute for Neurological Diseases, Gainesville, FL, USA; ^3^ Department of Clinical and Health Psychology (B.M.A.), University of Florida, Gainesville, FL, USA; ^4^ 1Florida Alzheimer's Disease Research Center, Gainesville, FL, USA; ^5^ University of Florida Center for Cognitive Aging and Memory, Gainesville, FL, USA; ^6^ Florida Alzheimer's Disease Research Center, Gainesville, FL, USA

## Abstract

**Background:**

Alzheimer's disease (AD) and Lewy body (LB) pathology frequently co‐occur. While α‐synuclein accumulation characterizes LB pathology, its impact on brain aging, atrophy, and function when combined with AD remains poorly understood. Advances in α‐synuclein seed amplification assays (SAA) enable in vivo detection of LB, offering new insights into its interplay with AD.

**Method:**

We leveraged structural MRI data from cognitively unimpaired individuals across five cohorts (NACC, ADNI, HCP, CamCAN, AIBL; *n* = 4,355) to train a 3D‐DenseNet deep learning (DL) model for brain age estimation. In a separate cohort of cognitively impaired participants from ADNI (*n* = 803), we used CSF SAA to determine α‐synuclein positivity and the *p*‐tau_181_/Aβ_42_ ratio to define AD positivity, classifying participants into four pathology subgroups: AD‐LB‐, AD‐LB+, AD+LB‐, and AD+LB+. We then employed our trained DL model and longitudinal mixed‐effects analyses to comprehensively investigate their brain age gaps (a proxy for neurodegeneration), region‐specific atrophy, and multiple cognitive measures.

**Result:**

As shown in Figure 1, the trained DL model robustly captured normal aging trajectories in cognitively unimpaired individuals in ADNI (average brain age gap 0.31 ± 0.11 years; *r* ≈ 0.93). Among the four pathology subgroups, AD+LB+ displayed the greatest deviation in brain age gap, significantly surpassing either pathology alone (both *p* < 0.001), reflecting a further acceleration of brain aging. Sex‐stratified analyses revealed that males in AD‐ subgroups had higher brain age gaps than females, whereas in AD+ subgroups, females showed higher brain age gaps than males, suggesting sex‐dependent vulnerability. Figure 2 illustrates the DL‐driven saliency maps, highlighting more pronounced neurodegeneration in AD+LB+, aligning with its steeper longitudinal brain age gap progression (Figure 3A). Specifically, the maps show heightened saliency in the medial temporal, occipital, and basal ganglia regions for AD+LB+, corresponding to its more pronounced longitudinal atrophy in these regions (Figure 3B). Cognitively, AD+LB+ showed the most severe deficits in global and domain‐specific tests, following accelerated declines that outpaced single‐pathology subgroups (Figure 3C and D).

**Conclusion:**

These findings underscore α‐synuclein's synergistic role in amplifying AD‐related neurodegeneration, highlighting the importance of combined biomarker assays and targeted interventions for individuals harboring co‐existing AD and LB pathology.